# PaCO2 Association with Traumatic Brain Injury Patients Outcomes at High Altitude: A Prospective Single-Center Cohort Study

**DOI:** 10.21203/rs.3.rs-3876988/v1

**Published:** 2024-01-24

**Authors:** Eder Caceres, Afshin A. Divani, Clio A. Rubinos, Juan Olivella-Gómez, André Emilio Viñán-Garcés, Angélica González, Alexis Alvarado-Arias, Kunal Bathia, Uzma Samadani, Luis F. Reyes

**Affiliations:** Universidad de La Sabana; University of New Mexico - Albuquerque: The University of New Mexico; University of North Carolina at Chapel Hill Health Sciences Library: The University of North Carolina at Chapel Hill; Universidad de La Sabana; Universidad de La Sabana; Universidad de La Sabana; University of Mississippi University Hospital: The University of Mississippi Medical Center; University of Mississippi University Hospital: The University of Mississippi Medical Center; Minneapolis VAMC: Minneapolis VA Medical Center; Universidad de La Sabana

**Keywords:** traumatic brain injury, neurocritical care, carbon dioxide, mechanical ventilation, disability, head injury, trauma, outcomes, high altitude

## Abstract

**Background:**

partial pressure of carbon dioxide (PaCO2) is generally known to influence outcome in patients with traumatic brain injury (TBI) at normal altitudes. Less is known about specific relationships of PaCO2 levels and clinical outcomes at high altitudes.

**Methods:**

This is a prospective single-center cohort of consecutive TBI patients admitted to a trauma center located at 2600 meter above sea level. An unfavorable outcome was defined as the Glasgow Outcome Scale-Extended (GOSE) < 4 at 6-month follow-up.

**Results:**

81 patients with complete data, 80% (65/81) were men, and median (IQR) age was 36 (25–50) years). Median Glasgow Coma Scale (GCS) on admission was 9 (6–14), 49% (40/81) were severe (GCS: 3–8), 32% (26/81) moderate (GCS 12 − 9), and 18% (15/81) mild (GCS 13–15) TBI. The median (IQR) Abbreviated Injury Score of the Head (AISh) was 3 (2–4). Frequency of an unfavorable outcome (GOSE < 4) was 30% (25/81), median GOSE was 4 (2–5), and 6-month mortality was 24% (20/81). Comparison between patients with favorable and unfavorable outcomes revealed that those with unfavorable outcome were older, median [49 (30–72) vs. 29 (22–41), P < 0.01], had lower admission GCS [6 (4–8) vs. 13 (8–15), P < 0.01], higher AIS head [4 (4–4) vs. 3(2–4), p < 0.01], higher APACHE II score [17(15–23) vs 10 (6–14), < 0.01), higher Charlson score [0(0–2) vs. 0 (0–0), P < 0.01] and higher PaCO2 (mmHg), mean ± SD, 39 ± 9 vs. 32 ± 6, P < 0.01. In a multivariate analysis, age (OR 1.14 95% CI 1.1–1.30, P < 0.01), AISh (OR 4.7 95% CI 1.55–21.0, P < 0.05), and PaCO2 (OR 1.23 95% CI: 1.10–1.53, P < 0.05) were significantly associated with the unfavorable outcomes. When applying the same analysis to the subgroup on mechanical ventilation, AISh (OR 5.4 95% CI: 1.61–28.5, P = 0.017) and PaCO2 (OR 1.36 95% CI: 1.13–1.78, P = 0.015) remained significantly associated with the unfavorable outcome.

**Conclusion:**

Higher PaCO2 levels are associated with an unfavorable outcome in ventilated TBI patients. These results underscore the importance of PaCO2 level in TBI patients and whether it should be adjusted for populations living at higher altitudes.

## INTRODUCTION

Traumatic Brain Injury (TBI) accounts for a substantial global health burden, with approximately 27 million cases reported annually, particularly in low- and middle-income countries (LMICs) (1, 2). As many as 50% of individuals with TBI do not regain their previous functionality (3), resulting in a reported age-standardized incidence rate of 111 (82–141) years lived with disability per 100,000 (4). The most frequently cited factors related to poor outcomes include age, trauma severity, and the Glasgow Coma Scale (GCS) at presentation. Other factors, such as imaging findings, hypoxia, hypo or hypercapnia, and hypotension, have also been identified. (5, 6, 7). These findings have allowed clinical teams and guidelines to establish goals in the acute setting to optimize care to limit secondary brain injury. These goals often include specific hemodynamic and respiratory parameters to achieve a particular target, such as optimal levels of partial pressure of carbon dioxide (PaCO2) (8, 9).

**Figure 1:**
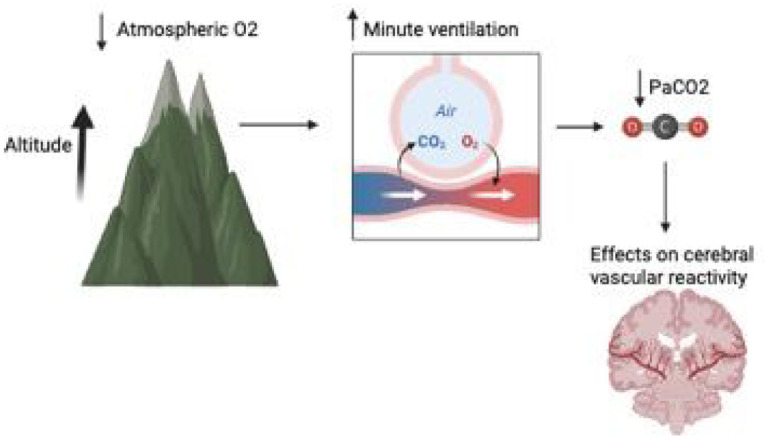
Effect of altitude on ventilation and cerebral vascular reactivity. Lower atmospheric pressure at a higher altitude leads to a compensatory increase in the minute ventilation which reduce the PaCO2. How a lower baseline of PaCO2 affects the cerebral vasoreactivity, especially in TBI, and its therapeutic implications needs further investigation.

Carbon dioxide plays a central role in regulating cerebral blood flow, a notion supported by animal and human studies (10). Hypercapnia causes blood vessels to dilate due to cerebrospinal fluid acidosis and the direct effect of extracellular H + on vascular smooth muscle (11), while hypocapnia constricts them via alkalosis, influencing intracranial pressure and adjusting brain tissue perfusion in response to the environment (12). Maintaining optimal partial pressure of carbon dioxide (PaCO2) levels is crucial in cases of brain injury, as hypoperfusion and hypoxemia are closely linked to secondary brain injury and long-term consequences, impacting disability and survival rates (13, 14). Guidelines recommend maintaining a target PCO2 range between 35–45 mm Hg to prevent cerebral ischemia in the case of low PaCO2 or hyperemia that could lead to elevated intracranial pressure if PaCO2 is high (15). Several studies have reinforced this concept of targeting a specific range of PaCO2 as a goal of care for TBI patients in the neuro-intensive care unit (NICU) (16) and its potential systemic implications (17, 18). There is also considerable variability in the management of PaCO2 in TBI patients within regions and centers (19). Furthermore, evidence indicates that normal PaCO2 levels can vary according to altitude and barometric pressure (20, 21). Generally, the barometric pressure is 760 mmHg at sea level, with PaCO2 levels between 35–45 mmHg considered normal (21). At higher altitudes, the atmospheric pressure of O2 and CO2 is lower, reducing PaO2 and PaCO2 (alveolar pressure), which in turn stimulates alveolar ventilation (22, 23). The implications of these differences on the physiology and management of patients with TBI are unclear. Further contributions in this area may help guide the management and care of this patient population (Fig. 1).

We hypothesize that the TBI population at higher altitudes may benefit from different PaCO2 level targets compared to sea-level populations. Additionally, we hypothesized that the initial management of respiratory care and support in the acute phase might influence outcomes. This study evaluates the association between admission PaCO2 levels and outcomes at 6-month follow-ups in patients with TBI admitted to the NICU.

## MATERIALS AND METHODS

The study was approved by the Institutional Review Board/Independent Ethics Committee (IRB/EC) under the local regulations and the Declaration of Helsinki for clinical practices, including obtaining informed consent from the patient representative. All clinical data were anonymized and collected using the Research Electronic Data Capture (REDCap), an electronic data collection form provided by the Universidad de La Sabana.

### Study Population

This single-center prospective cohort study was conducted in a trauma center at the Universidad de La Sabana in Chía, Colombia. We consecutively recruited and collected data from TBI patients admitted to the NICU from December 2019 to June 2022. The diagnosis, inclusion, and exclusion criteria, as well as imaging studies, were obtained by chart review.

The study cohort included ≥ 18-year-old TBI patients admitted to the NICU within 24 hours post-injury and who stayed in the NICU for more than 48 hours. Patients with a previous history of disability or debilitating diseases measured by a modified Rankin Scale (mRS) > 2 and those admitted after 24 hours post-injury were excluded.

### Definitions

To evaluate the severity of TBI, we utilized the Abbreviated Injury Scale of the head (AISh). We chose to use AISh because GCS was often obscured by sedation at the injury site or upon admission to the emergency department (ED). AISh incorporates both clinical and imaging findings (24, 25), enabling a more nuanced assessment of the severity of the lesion (Table S1) and provides a robust correlation with outcomes. The AIS ranks injury on an ordinal scale 0 to 6 (from no injury to fatal). AIS can be classified as 1 (minor injury), 2 (moderate), 3 (serious), 4 (severe), 5 (critical) or 6 (fatal) (26, 27).

To assess the severity of the traumatic injury overall, the injury severity score (ISS) was used. The ISS is a composite measure derived from the AIS score that includes a rating of the three most severely injured body regions and ranges from 0 to 75. An ISS of 15 or higher is usually considered major trauma, and the compromise of 2 or more body regions with an AIS ≥ 3 is considered multiple trauma (27).

To characterize the severity of the brain injury in a head CT scan, we used the Marshall classification. The Marshall scheme was first published in 1992 and uses six categories (I to VI) of increasing severity based on non-contrast CT scan findings, including midline shift, compression of cisterns, and mass lesions (28, 29) (Table S2). Its correlation with outcomes in TBI has been validated in several studies (30, 31)

The International Mission for Prognosis and Analysis of Clinical Trials in TBI (IMPACT) is a prognostic model that uses baseline characteristics and provides a probability of an unfavorable outcome and mortality at 6 months (Table S3). It defines an unfavorable outcome as a Glasgow Outcome Scale of 1–3. The IMPACT model has accurately discriminated outcomes after TBI (32, 33). We used the lab model that includes age, motor score of the GCS, pupillary reactivity, CT characteristics, and information on admission hemoglobin and glucose. (33).

To evaluate mortality and disability as outcomes, we selected the Glasgow Outcome Scale-Extended (GOSE), as outlined in Table S4, which is an ordinal scale of eight points ranging from death to good recovery (34). GOSE has been used widely to assess outcomes in TBI (35, 36, 37). A trained staff administered GOSE through a standardized phone interview with the patients or their caregivers 6 months post-injury. For the analysis, we dichotomized GOSE into favorable and unfavorable outcomes. A favorable outcome (GOSE ≥ 4) was considered for those with upper severe disability to upper good recovery, and an unfavorable outcome was defined as a lower severe disability to death (GOSE < 4).

Infectious complications were evaluated using the Infectious Disease Society of America/ American Thoracic Society guidelines definitions, including Ventilator-associated Pneumonia (VAP), Ventilator-associated Tracheitis (VAT), Catheter-Associated Urinary Tract Infection (CAUTI), Surgical Site Infection, and Catheter-Related Bloodstream Infection (38, 39, 40, 41).

### Data Collection

Upon admission to the NICU, demographic data and trauma severity and prognostication scales that include the GCS, ISS, IMPACT model, and Marshall CT scan classification were recorded consecutively and prospectively. We collected admission vital signs and lab tests, which were reviewed and confirmed directly from the electronic medical record. Medical interventions during NICU stay, including mechanical ventilation, blood components transfusion, and use of vasopressors within 72 h of admission were reported. Finally, infections in the NICU, total hospital and NICU length-of-stay (LOS), and hospital mortality were also recorded. At 6-month follow-up, patients or their legal representatives were contacted via phone by a trained research team member to administer GOSE.

### Statistical Analysis

Continuous variables were summarized based on clinical relevance and distribution using minimum and maximum values, mean ± standard deviation (SD), or median and interquartile range (IQR). Dichotomous variables were presented as frequencies and percentages. Differences between intervention groups were assessed by applying the chi-square and Fisher’s exact tests for categorical variables. In contrast, continuous variables were evaluated using the student’s t-test or Mann-Whitney U test, depending on their distribution.

A multivariate logistic regression model was constructed for the general cohort to investigate the risk factors associated with unfavorable outcomes at 6-month follow-up. The model was adjusted for admission demographic data, vital signs, and lab tests. The logistic regression used the best subset method for the variable selection and included variables with a P value of less than 0.10 in the univariate analysis. Odds ratios (OR) with a 95% confidence interval (95% CI) were calculated based on the exponential values of the coefficients obtained from the final model D. We used R Studio (Version 2023.09.1 + 494) for the analysis.

## RESULTS

### Patient Demographics and Characteristics

From December 2019 to June 2022, 81 TBI patients admitted to the NICU at La Sabana Hospital in Chia, Colombia, located at 2600 meters above sea level, were included in the study. The baseline and clinical characteristics are presented in Table 1. The median (IQR) age was 36 (25–50) years, men accounted for 80% (n = 65) of the population. Traffic accidents were the leading cause of injury (60%, 49/81), followed by falls (24%, 19/81), cycling (9%, 8/81), violence (4%, 3/81), and others (3%, 2/81). Isolated TBI was present in 32% (26/81), and the most associated injuries were thorax 37% (30/81), limbs 20% (17/81), and abdomen 18% (14/81). Median (IQR) GCS on admission was 9 (6–14). The severity of TBI according to the AISh was moderate (AISh 2) in 27% (22/81), serious (AIS 3) in 27% (22/81), severe (AIS 4) in 33% (27/81), and critical (AIS 5) in 13% (10/81). The median (IQR) for AISh was 3 (2–4). In terms of the overall severity of trauma, the median (IQR) of ISS was 24 (13–32), among which 72% (58/81) had major trauma (ISS > 15). Structural severity of head trauma was determined through the Marshall CT classification, where 31% (24/81) of cases fell into diffuse injury I, 36% (31/81) diffuse injury II, 8% (6/80) diffuse injury III, 20% (16/81) evacuated mass lesion V, 4% (3/81) non-evacuated mass lesion VI, and 1% (1/81) diffuse injury IV. The most frequent primary visible injury on head CT was contusion in 61% (50/81), followed by traumatic subarachnoid hemorrhage (tSAH) 37% (30/81), subdural hematoma 29% (24/81), epidural hematoma 24% (20/81) and diffuse axonal injury 11% (9/81). All patients were admitted to the NICU, 60% (49/81) on invasive mechanical ventilation. The NICU and hospital LOS were 6 (4–15) and 11 (6–23) days, respectively. Tracheostomy and gastrostomy were performed in 20% (16/81) and 15% (12/81) of the cases, respectively. The tracheostomy procedure was performed 10 (7–14) days post-admission. At least one infectious complication was diagnosed in 30% (25/81) of patients during their NICU stay. Out of the patients that had an infection, the sources of infection were VAP in 28% (7/25), VAT in 64% (16/25), CAUTI in 16% (4/25), one case of surgical site infection, and one case of sinusitis. When applying the IMPACT lab model to the entire cohort, the median (IQR) probability of a 6-month unfavorable outcome was 16% (7–38).

### Outcome

At 6 months post-injury, we were able to conduct phone interviews with all survivors or their caregivers to administer GOSE (n = 71). Patients who died during the hospitalization (n = 10, 12%) were included in the unfavorable outcome group. A total of 56 patients had a favorable outcome (GOSE: 4–8) and 25 patients had an unfavorable outcome (GOSE: 1–3). The frequency of an unfavorable outcome (GOSE < 4) was 30% (25/81) at 6 months. The median (IQR) GOSE at 6 months was 4 (2–5). Mortality at 6 months was 24% (20/81).

**Figure 2:**
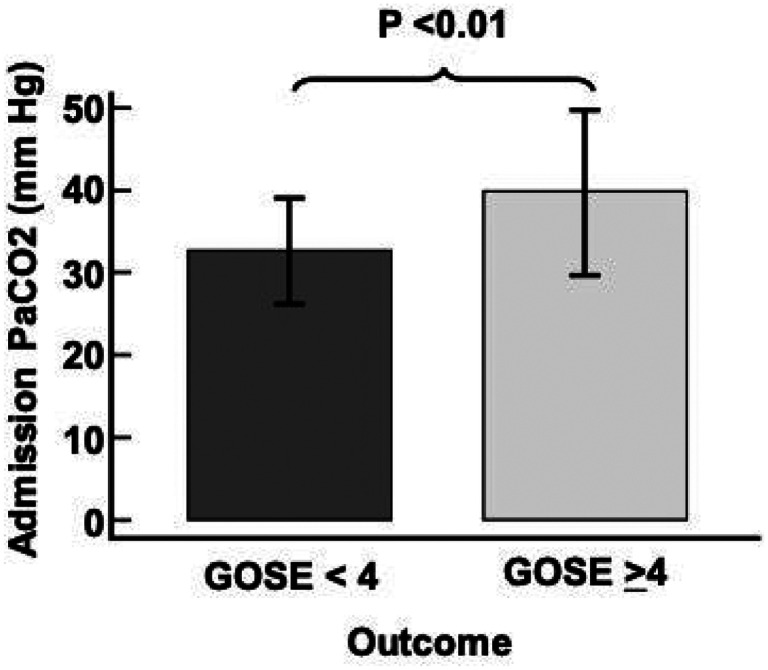
Admission PaCO2 levels (mean ± SD) for the 6-month outcome for TBI patients. Glasgow Outcome Scale-Extended (GOSE) for unfavorable outcome (< 4) and favorable outcome (>4) outcomes.

Comparison between patients with a 6-month favorable outcome and unfavorable outcome (Table 1) revealed that those with an unfavorable outcome were older [49 (30–72) vs. 29 (22–41) years, P < 0.01], had a lower admission GCS [6(4–8) vs. 13 (8–15), P < 0.01], a higher AISh [4 (4–4) vs. 3(2–4), P < 0.01], increased probability of a poor outcome by the IMPACT model (%) [43 (31–64) vs. 13 (6–17), P < 0.01], higher APACHE II score [17 (15–23) vs. 10 (6–14), P < 0.01), higher Charlson score [0 (0–2) vs. 0 (0–0), P < 0.01] and higher PaCO2 [39 ± 9 vs. 32 ± 6 mm Hg, P < 0.01, Fig. 2]. In terms of hospital variables and interventions, the group with an unfavorable 6-month outcome was more frequently on mechanical ventilation (88% [22/25] vs. 41% (27/56), P < 0.01) and it required vasopressors in 84% (21/25) vs. 48% (24/56) of cases, P < 0.01. The group with the unfavorable outcomes also required neurosurgical intervention, 60% (15/25) vs. 9% (5/56), and it underwent tracheostomy in a greater proportion during NICU stay [48% (12/25) vs. 4% (2/56)]. Other data collected on admission include systolic blood pressure (SBP), heart rate (HR), respiratory rate (RR), white blood cell count (WBC), platelet count, serum sodium, lactate, PaO2, hemoglobin, and serum glucose; none of these variables were different between the groups with favorable and unfavorable outcomes (Table 1).

### PaCO2 and Outcome for Patients on Mechanical Ventilation

**Figure 3:**
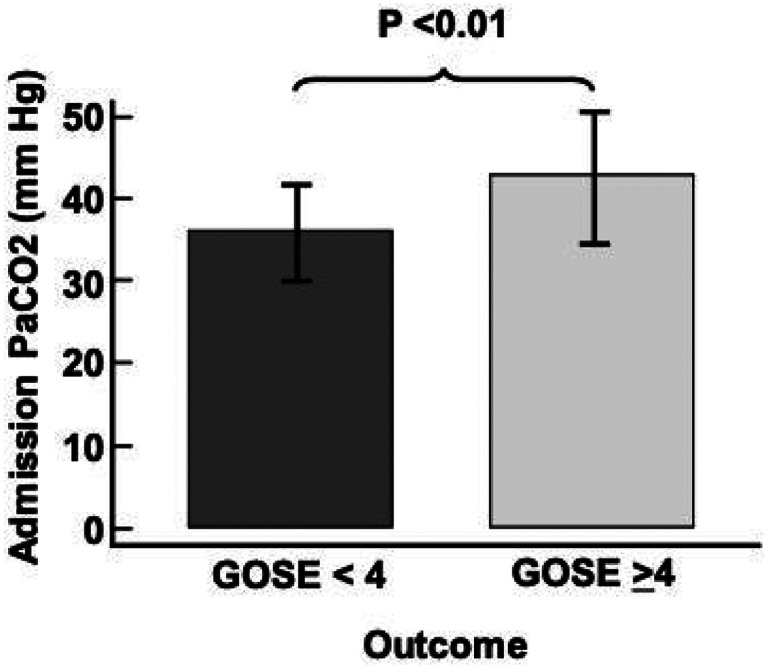
Admission PaCO2 levels (mean ± SD) for the 6-month outcome for TBI patients admitted to the NICU on mechanical ventilation. Glasgow Outcome Scale-Extended (GOSE) for unfavorable outcome (< 4) and favorable outcome (≥4) outcomes.

When evaluating the group on mechanical ventilation (n = 49), the PaCO2 mean ± SD was 39.0 ± 7.7 mmHg, which was significantly higher for those with a 6-month unfavorable outcome compared to the group with a favorable outcome (42.0 ± 7.8 vs. 35.3 ± 4.4, P < 0.01, Fig. 3). In the group without ventilatory support, the PaCO2 mean ± SD was 28.1 ± 5.8 mmHg, and it was significantly lower for the group with an unfavorable outcome (21.6 ± 2.5 vs 28.9 ± 5.6, P < 0.01) compared to those with favorable outcome at 6 months. Mean PaCO2 was lower in the group without ventilator support than those on mechanical ventilation (28.1 ± 5.8 vs. 39.0 ± 7.7, P < 0.001). Finally, NICU LOS was longer for the unfavorable outcome group, 14 days (6–23) vs. 5 days (4–8), P < 0.01.

### Logistic regression analysis

Univariate analysis of in-hospital variables and their association with a 6-month unfavorable outcome were performed through a univariate logistic regression (P < 0.1) (Table 2). Variables significantly associated with the primary outcome included age (OR 1.01, 95% CI: 1.01–1.02), GCS (OR 1.60, 95% CI: 1.30–2.10), AIS head (OR 1.21, 95% CI: 1.11–1.32), use of vasopressors within 72 h of admission (OR 1.5, 95% CI: 1.20–1.80), mechanical ventilation (OR 1.4, 95% CI: 1.16–1.8), infectious complications (OR 1.45, 95% CI: 1.2–1.8), neurosurgical intervention (OR 1.6, 95% CI: 1.3–2.0), and need for a tracheostomy (OR 1.8, 95% CI: 1.4–2.3). Regarding the laboratory data on admission, the one with a significant association was PaCO2 (OR 1.02, 95% CI: 1.01–1.03). From the variables with a significant association in the univariate analysis (P < 0.1), age, AIS head, APACHE II, and PaCO2 were included in the multivariate analysis. From those, age (OR 1.14 95% CI: 1.1–1.30, P < 0.01), AIS head (OR 4.7, 95% CI: 1.55–21.0, P < 0.05), and PaCO2 (OR 1.23, 95% CI: 1.10–1.53, P < 0.05) remained significantly associated with the 6-month unfavorable outcome in the multivariate analysis (Table 2).

Afterward, the same analysis was applied to the subgroups of patients with and without ventilator support. A multivariate analysis was performed on the mechanical ventilation group using the same variables: age, AISh, APACHE II, and PaCO2. In this case, again, AIS head (OR 5.4, 95% CI: 1.61–28.5, P = 0.017) and PaCO2 (OR 1.36, 95% CI: 1.13–1.78, P = 0,015) remained significantly associated with the 6-month unfavorable outcome (Table 3). The same analysis for the group without mechanical ventilation did not yield a significant result for any of the variables (P = 1). Hosmer-Lemeshow test for binary logistic regression models demonstrated the goodness-of- fit test (P = 0.97).

## DISCUSSION

This study initially characterizes a prospective cohort of patients with TBI admitted to the NICU in an academic center in the Andean region in Colombia. The group with an unfavorable outcome was older, had lower GCS on admission, higher AISh, higher probability of an unfavorable outcome by the IMPACT-TBI model, higher APACHE II, and higher Charlson score. Among vital signs and laboratory data, the only documented difference was a higher PaCO2 on admission for those with an unfavorable outcome. In terms of in-hospital procedures, the group with an unfavorable outcome required more ventilatory and hemodynamic support, underwent neurosurgical interventions and tracheostomy more often, and had a longer LOS in the NICU. After adjusting for age, severity of TBI, and APACHE II, PaCO2 remained directly correlated with an unfavorable outcome at 6 months. A higher PaCO2 was associated with an unfavorable 6-month outcome for all the study groups and the group on ventilatory support. In the subgroup, without ventilatory support, this correlation was not maintained. The mean PaCO2 in the subgroup without ventilatory support was lower than those on mechanical ventilation. The lower PaCO2 levels observed in the non-ventilated group may be associated with the inherently lower baseline levels of PaCO2 in populations residing at higher altitudes. Consequently, this suggests a potential difference in the way regulatory mechanisms are established (10, 13).

The demographic characteristics of the studied cohort are similar to what others have found in terms of age and cause of trauma (42, 43). TBI affects predominantly the adult male population in their fourth or fifth decade of life, and the leading causes of injury are road accidents and falls. This has been consistent in several prospective studies, including the European and Chinese cohorts of CENTER-TBI and the TRACK-TBI for the US (5, 42, 44). Regarding mortality and functional outcomes, the ICU stratum of the European Center-TBI found 43.1% and 21.3% rates of an unfavorable outcome (GOSE < 5) and mortality, respectively. The results in our study are similar in both mortality (24%) and unfavorable outcome (30%), bearing in mind that the definition we used for unfavorable outcome was GOSE < 4 (44). There is no standardized manner to dichotomize GOSE, and definitions vary across studies (45, 46). TBI patients might show functional and cognitive improvement even 1 year after the trauma (47, 48), depending on their recovery trajectory. GOSE equal to 4 refers to a person who requires partial supervision and assistance but can be on their own at home for at least 8 hours a day. Therefore, we considered it reasonable to define GOSE ≥ 4 as the favorable outcome, considering that those patients are already partially independent at home and still have the potential for further progress.

Several studies have pointed out that older and more severely injured TBI patients have more frequent severe disability and functional dependence after TBI (49, 50). Moderate and severe TBI cases are usually admitted to the NICU, where interventions are guided by targets that aim to protect the brain from a secondary injury (51). Henceforth, it is also the more severely traumatized patient who needs more assistance in terms of respiratory, hemodynamic, and metabolic support as well as surgical interventions (52, 53). In our cohort, the group with unfavorable outcomes was older and had a more severe TBI on admission. Therefore, it could be expected that it is, in turn, the group that received a higher burden of care, including mechanical ventilation, vasopressors, neurosurgical and tracheostomy procedures, and was more exposed to complications like in-hospital infections and longer ICU stays. This reflects the complexity of treatment and prognosis when many factors are involved, leaving aside the variability of management across centers and regions (53). Despite this challenge, some prognostic models have been developed and validated, for instance, The Corticosteroid Randomization After Significant Head Injury (CRASH) model and the International Mission for Prognosis and Analysis of Clinical Trials (IMPACT) in TBI model (54, 55, 56). These models estimate the probability of disability and mortality and consider factors such as age, Glasgow motor score, pupillary reactivity, and imaging findings on head CT scans. We did not intend to develop a model, but we did identify some factors on admission associated with outcomes, including age, severity of TBI, APACHE II, and need for hemodynamic and ventilatory support. However, when assessing vital signs and laboratory tests, higher levels of PaCO2 on admission were associated with the unfavorable outcome, even after controlling for the age and severity of the injury. The role of PaCO2 in this context relies on its effect on the cerebral vasculature or vasoreactivity (57, 58). The brain has high metabolic demand, requiring a constant supply of oxygen and glucose (59). This supply is ensured through a tightly regulated cerebral blood flow that matches each brain region’s temporal and spatial metabolic requirements (60). One of those mechanisms is the vasomotor response to carbon dioxide, where cerebral arterioles dilate or contract according to changes in PaCO2. This response has a sigmoidal shape and functions within the 20–60 mmHg of PaCO2. Every 1 mmHg increase in PaCO2 corresponds to roughly a 4% increase in cerebral blood flow (61, 62), which in turn increases the cerebral blood volume resulting in an intracranial pressure elevation and finally affecting the cerebral perfusion pressure. Several cohorts have demonstrated the effect of PaCO2 management on outcomes, including mortality (21). However, variability in management exists across centers (63). Guidelines recommend a normal range ventilation, PaCO2 35–45 mmHg, and avoidance of hyperventilation and severe (< 25 mmHg) or moderate (< 30 mmHg) hypocapnia (8, 9) given the risk of brain ischemia.

In our cohort, we found a higher PaCO2 for those cases with an unfavorable outcome, and the multivariate analysis revealed a direct relation between admission PaCO2 levels and the probability of death and disability. The association remained for the subgroup on mechanical ventilation but not for those patients without ventilatory support. This could be expected given that PaCO2 in a ventilated patient depends mostly on the ventilator settings and can be adjusted to a specific goal. However, we would like to point out that most of our patients had PaCO2 levels within the recommended range of 35–45 mmHg and even below for those with a favorable outcome, 32 ± 6 mmHg. In addition, non-ventilated patients had even lower PaCO2 levels. These results underscore the importance and impact of PaCO2 as a crucial target in the management of ventilated TBI patients and raise the question of whether, for populations at higher altitudes, different PaCO2 goals should be pursued. Further investigation would be needed to answer this question, which will benefit a substantial proportion of the global TBI population living at higher altitudes.

Limitations of our study include a single-center study that requires further validation to make the results more generalizable. In addition, we only recorded the admission PaCO2 values rather than serial values.

## CONCLUSION

We evaluated the relationship between PaCO2 levels and functional outcomes in patients with TBI admitted to the ICU. Interestingly, in our center, situated at a higher altitude above sea level, we observed that in the sample of patients on mechanical ventilation, a PaCO2 below the recommended target was associated with improved outcomes. Although this is a single-center prospective cohort study, it raises the question of whether the target PaCO2 levels need adjustment in populations at higher altitudes.

## Figures and Tables

**Table 1:** Baseline characteristics of patients admitted to NICU for TBI and comparison between groups with favorable and unfavorable outcomes.

	Overall	Favorable outcome	Unfavorable outcome	P value
	(n = 81)	(n = 56)	(n = 25)	
Age, median (IQR)	36 (25–50)	29 (22–41)	49 (30–72)	**<0.01**
Sex male n (%)	65 (80)	34 (70)	23 (92)	0.18
Admission GCS, median (IQR)	9 (6–14)	13 (8–15)	6 (4–8)	**<0.01**
ISS, median (IQR)	24 (13–32)	27 (20–34)	22 (12–29)	0.052
AIS head median (IQR)	3 (2–4)	3 (2–4)	4 (4–4)	**<0.01**
IMPACT-TBI outcome (%), median (IQR)	16 (7–38)	13 (6–17)	43 (31–64)	**<0.01**
Marshall Classification head CT n (%)	DI I: 24 (31)	DI I 22 (39)	DI I 2 (8)	**0.009**
	DI II: 31 (36)	DI II 26 (46)	DI II 5 (20)	**0.044**
	DI III: 6 (8)	DI III 2 (4)	DI III 4 (16)	0.1
	DI IV: 1 (1)	DI IV 0 (0)	DI IV 1 (4)	0.6
	EML V: 16 (20)	EML V: 4 (7)	EML V 12 (48)	**< 0.001**
	NEML VI: 3 (4)	NEML VI: 2 (3)	NEML 1 (4)	
APACHE II, median (iQr)	12 (7–17)	10 (6–14)	17 (15–23)	**<0.01**
Charlson score, median (IQR)	0 (0–0)	0 (0–0)	0 (0–2)	**<0.01**
Admission SBP,mean ± SD	122 ±20	119 ± 19	128 ± 24	0.09
Admission Heart rate, mean ± SD	91 + 21	91 + 20	84 + 27	0.27
Admission Respiratory rate, median (IQR)	20 (18–22)	19 (18–21)	20 (18–22)	0.3
Admission WBC × 10^3^/dl mean ± SD	16 ± 5	16,9 ± 4	15.1 (5,4)	0.16
Admission Hemoglobin, median (IQR), grs/dl	14 (12–15)	14,2 (12,7–14,9)	14 (12–15)	0.4
Admission Platelets, mean ± SD	231 ±79	240 ±88	213 ± 66	0.15
Serum sodium, median (IQR), mEq/L	139 (137–142)	140 (138–141)	139 (137–142)	0.38
PaO2, median (IQR), mmHg	85 (71–123)	86 (70–127)	88 (72–124)	0.7
PaCO2 mmHg, mean ± SD	35 ± 8	32 ±6	39 ±9	**<0.01**
Serum lactate, median (IQR), mmol/L	2.7 (1,8 – 3,9)	2.7 (1,9 – 3,8)	2.8 (1,8 – 4,3)	0.6
Serum glucose, median (IQR), mg/dl	130 (120–160)	130 (120–160)	140 (120–160)	0.3
Mechanical ventilation n (%)	49 (60)	27 (49)	22 (88)	**0.003**
Days on mechanical ventilation, median (IQR)	6 (3–12)	3 (2–8)	10 (4–18)	**0.008**
Vasopressors within 72h n (%)	45 (55)	24 (44)	21 (84)	**0.002**
Neurosurgical intervention n (%)	20 (24%)	5 (9)	15 (60)	**<0.01**
Infectious complication n (%)	25 (30)	9 (20)	14 (56)	**0.004**
Tracheostomy n (%)	16 (20)	2 (4)	12 (48)	**<0.01**
NICU LOS, median (iQr), days	6 (4–15)	5 (4–8)	14 (6–23)	**0.002**
Hospital LOS, median (IQR), days	11 (6–23)	11 (6–13)	22 (7–43)	0.06

Abbreviations: AIS head: Abbreviated Injury Score of the head, DI I: Diffuse Injury I, DI II: Diffuse Injury II, DI III: Diffuse Injury III, DI IV: Diffuse Injury IV, EML V: Evacuated Mass Lesion V, GCS: Glasgow Coma Scale, IMPACT TBI: International Mission for Prognosis and Analysis of Clinical Trials in Traumatic Brain Injury, ISS: Injury Severity Score, NEML VI: Non.-evacuated Mass Lesion VI, NICU: Neuro-Intensive Care Unit, SBP: Systolic Blood Pressure, WBC: White Blood Cell Count.

**Table 2:** Univariate and multivariate analysis of variables on admission and a 6-month unfavorable outcome in patients with TBI admitted to NICU.

	Univariate Analysis		Multivariate Analysis	
Variables	OR (95% CI)	P value	OR (95% CI)	P value
**Demographics**				
Age	1.01 (1.01–1.02)	**<0.01**	1.14 (1.10–1.30)	**<0.01**
**Severity of injury**				
Admission GCS	1.60 (1.30–2.10)	**<0.01**		
AIS head	1.21 (1.11–1.32)	**<0.01**	4.7 (1.55–21.0)	**0.016**
APACHE II	1.04 (1.03–1.05)	**<0.01**	0.90 (0.70–1.30)	0.90
**Medical interventions**				
Vasopressor requirement*	1.50 (1.20–1.80)	**<0.01**		
Transfusion of blood components*	1.20 (0.90–1.50)	0.14		
Invasive mechanical ventilation	1.40 (1.16–1.80)	**<0.01**		
**Labs on admission**				
Leucocytes, cell/mm10^3^	0.98 (0.95–1.01)	0.13		
Hemoglobin, g/dL	0.97 (0.92–1.02)	0.30		
Platelets, cell/mm^3^	0.99 (0.99–1.00)	0.18		
Serum sodium, mEq/L	0.99 (0.97–1.00)	0.90		
PaO2, mmHg	1.00 (0.99–1.00)	0.80		
PaCO2, mmHg	1.02 (1.01–1.03)	**<0.01**	1.23 (1.10–1.53)	**0.026**
Lactic acid, mmol/L	1.00 (0.95–1.03)	0.50		
Glucose, mgr/dL	1.00 (0.99–1.00)	0.19		
BUN	1.00 (0.97–1.02)	0.80		
Creatinine	1.20 (0.80–1.70)	0.20		

* Within 72 hours of admission

GCS: Glasgow Coma Scale; AIS: Abbreviated Injury Score; BUN: blood ureic nitrogen.

**Table 3:** Multivariate analysis of clinical variables and a 6-month unfavorable outcome in TBI patients admitted to the NICU on mechanical ventilation.

	Multivariate Analysis	
Variables	OR (96% CI)	P value
Age	1.09 (1.01–1.24)	0.06
Apache II	1.2 (0.98–1.59)	0.10
AIS head	5.4 (1.61–28.5)	**0.017**
PaCO2 on admission	1.36 (1.13–1.78)	**0.015**

AIS: Abbreviated Injury Score

## KEY POINTS

- PaCO2 management is crucial to treating patients with TBI on mechanical ventilation, considering its impact on clinical outcomes.
- The goal of PaCO2 in managing TBI in populations at higher altitudes above sea level might differ.
- Further investigation is needed to answer this question which would contribute to optimizing the treatment of TBI patients on ventilatory support.

## References

[1] GBD 2016 Traumatic Brain Injury and Spinal Cord Injury Collaborators. Global, regional, and national burden of traumatic brain injury and spinal cord injury, 1990–2016: a systematic analysis for the Global Burden of Disease Study 2016. Lancet Neurol. 2019;18(1):56–87. doi: 10.1016/S1474-4422(18)30415-0. Epub 2018 Nov 26. Erratum in: Lancet Neurol. 2021;20(12):e7. 30497965 PMC6291456

[2] Meyfroidt G, Bouzat P, Casaer MP, Chesnut R, Hamada SR, Helbok R, Hutchinson P, Maas AIR, Manley G, Menon DK, Newcombe VFJ, Oddo M, Robba C, Shutter L, Smith M, Steyerberg EW, Stocchetti N, Taccone FS, Wilson L, Zanier ER, Citerio G. Management of moderate to severe traumatic brain injury: an update for the intensivist. Intensive Care Med. 2022;48(6):649–666. 10.1007/s00134-022-06702-4. Epub 2022 May 20. Erratum in: Intensive Care Med. 2022;48(7):989–991. 35595999

[3] Lo J, Chan L, Flynn S. A Systematic Review of the Incidence, Prevalence, Costs, and Activity and Work Limitations of Amputation, Osteoarthritis, Rheumatoid Arthritis, Back Pain, Multiple Sclerosis, Spinal Cord Injury, Stroke, and Traumatic Brain Injury in the United States: A 2019 Update. Arch Phys Med Rehabil. 2021;102(1):115–31. Epub 2020 Apr 24. 32339483 10.1016/j.apmr.2020.04.001 PMC8529643

[4] GBD 2016 Traumatic Brain Injury and Spinal Cord Injury Collaborators. Global, regional, and national burden of traumatic brain injury and spinal cord injury, 1990–2016: a systematic analysis for the Global Burden of Disease Study 2016. Lancet Neurol. 2019;18(1):56–87. doi: 10.1016/S1474-4422(18)30415-0. Epub 2018 Nov 26. Erratum in: Lancet Neurol. 2021;20(12):e7. 30497965 PMC6291456

[5] Gao G, Wu X, Feng J, Hui J, Mao Q, Lecky F, Lingsma H, Maas AIR, Jiang J, China CENTER-TBI, Registry Participants. Clinical characteristics and outcomes in patients with traumatic brain injury in China: a prospective, multicentre, longitudinal, observational study. Lancet Neurol. 2020;19(8):670–677. 10.1016/S1474-4422(20)30182-4. 32702336

[6] Launey Y, Coquet A, Lasocki S, Dahyot-Fizelier C, Huet O, Le Pabic E, Roquilly A, Seguin P. Factors associated with an unfavourable outcome in elderly intensive care traumatic brain injury patients. a retrospective multicentre study. BMC Geriatr. 2022;22(1):1004. 10.1186/s12877-022-03651-x. 36585608 PMC9801582

[7] Carney N, Totten AM, O’Reilly C, Ullman JS, Hawryluk GW, Bell MJ, Bratton SL, Chesnut R, Harris OA, Kissoon N, Rubiano AM, Shutter L, Tasker RC, Vavilala MS, Wilberger J, Wright DW, Ghajar J. Guidelines for the Management of Severe Traumatic Brain Injury, Fourth Edition. Neurosurgery. 2017;80(1):6–15. 10.1227/NEU.0000000000001432. 27654000

[8] Picetti E, Rossi S, Abu-Zidan FM, Ansaloni L, Armonda R, Baiocchi GL, Bala M, Balogh ZJ, Berardino M, Biffl WL, Bouzat P, Buki A, Ceresoli M, Chesnut RM, Chiara O, Citerio G, Coccolini F, Coimbra R, Di Saverio S, Fraga GP, Gupta D, Helbok R, Hutchinson PJ, Kirkpatrick AW, Kinoshita T, Kluger Y, Leppaniemi A, Maas AIR, Maier RV, Minardi F, Moore EE, Myburgh JA, Okonkwo DO, Otomo Y, Rizoli S, Rubiano AM, Sahuquillo J, Sartelli M, Scalea TM, Servadei F, Stahel PF, Stocchetti N, Taccone FS, Tonetti T, Velmahos G, Weber D, Catena F. WSES consensus conference guidelines: monitoring and management of severe adult traumatic brain injury patients with polytrauma in the first 24 hours. World J Emerg Surg. 2019;14:53. 10.1186/s13017-019-0270-1. 31798673 PMC6884766

[9] Geeraerts T, Velly L, Abdennour L, Asehnoune K, Audibert G, Bouzat P, Bruder N, Carrillon R, Cottenceau V, Cotton F, Courtil-Teyssedre S, Dahyot-Fizelier C, Dailler F, David JS, Engrand N, Fletcher D, Francony G, Gergelé L, Ichai C, Javouhey É, Leblanc PE, Lieutaud T, Meyer P, Mirek S, Orliaguet G, Proust F, Quintard H, Ract C, Srairi M, Tazarourte K, Vigué B, Payen JF. ; French Society of Anaesthesia; Intensive Care Medicine; in partnership with Association de neuro-anesthésie-réanimation de langue française (Anarlf); French Society of Emergency Medicine (Société Française de Médecine d’urgence (SFMU); Société française de neurochirurgie (SFN); Groupe francophone de réanimation et d’urgences pédiatriques (GFRUP); Association des anesthésistes-réanimateurs pédiatriques d’expression française (Adarpef). Management of severe traumatic brain injury (first 24hours). Anaesth Crit Care Pain Med. 2018;37(2):171–186. 10.1016/j.accpm.2017.12.001. Epub 2017 Dec 27. 29288841

[10] Baron JC. The effect of changing arterial blood pressure and carbon dioxide on cerebral blood flow. J Neurol Neurosurg Psychiatry. 2020;91(7):678–9. 10.1136/jnnp-2019-322432. Epub 2020 Mar 25. 32213569

[11] Kontos HA, Raper AJ, Patterson JL. Analysis of vasoactivity of local pH, PCO2 and bicarbonate on pial vessels. Stroke. 1977 May-Jun;8(3):358 – 60. 10.1161/01.str.8.3.358. 16363

[12] Tague BW, Dickinson CD, Chrispeels MJ. A short domain of the plant vacuolar protein phytohemagglutinin targets invertase to the yeast vacuole. Plant Cell. 1990;2(6):533–46. 10.1105/tpc.2.6.533. 2152175 PMC159909

[13] Howard MB, McCollum N, Alberto EC, Kotler H, Mottla ME, Tiusaba L, Keller S, Marsic I, Sarcevic A, Burd RS, O’Connell KJ. Association of Ventilation during Initial Trauma Resuscitation for Traumatic Brain Injury and Post-Traumatic Outcomes: A Systematic Review. Prehosp Disaster Med. 2021;36(4):460–5. Epub 2021 May 31. 34057405 10.1017/S1049023X21000534 PMC8295185

[14] Bossers SM, Mansvelder F, Loer SA, Boer C, Bloemers FW, Van Lieshout EMM, Den Hartog D, Hoogerwerf N, van der Naalt J, Absalom AR, Schwarte LA, Twisk JWR, Schober P, BRAIN-PROTECT Collaborators. Association between prehospital end-tidal carbon dioxide levels and mortality in patients with suspected severe traumatic brain injury. Intensive Care Med. 2023;49(5):491–504. Epub 2023 Apr 19. 37074395 10.1007/s00134-023-07012-z PMC10205841

[15] Carney N, Totten AM, O’Reilly C, Ullman JS, Hawryluk GW, Bell MJ, Bratton SL, Chesnut R, Harris OA, Kissoon N, Rubiano AM, Shutter L, Tasker RC, Vavilala MS, Wilberger J, Wright DW, Ghajar J.Guidelines for the Management of Severe Traumatic Brain Injury, Fourth Edition. Neurosurgery. 2017;80(1):6–15. 10.1227/NEU.0000000000001432. 27654000

[16] Schirmer-Mikalsen K, Vik A, Skogvoll E, Moen KG, Solheim O, Klepstad P. Intracranial Pressure During Pressure Control and Pressure-Regulated Volume Control Ventilation in Patients with Traumatic Brain Injury: A Randomized Crossover trial. Neurocrit Care. 2016;24(3):332 – 41. 10.1007/s12028-015-0208-8. 26503512

[17] Haenggi M, Andermatt A, Anthamatten C, Galimanis A, Mono ML, Alfieri A, Fung C, Takala J, Jakob SM. CO(2)-Dependent vasomotor reactivity of cerebral arteries in patients with severe traumatic brain injury: time course and effect of augmentation of cardiac output with dobutamine. J Neurotrauma. 2012;29(9):1779–84. 10.1089/neu.2011.1809. Epub 2012 Apr 18. 21501044

[18] Wang YZ, Li TT, Cao HL, Yang WC. Recent advances in the neuroprotective effects of medical gases. Med Gas Res. 2019 Apr-Jun;9(2):80–7. 10.4103/2045-9912.260649. 31249256 PMC6607866

[19] Citerio G, Robba C, Rebora P, Petrosino M, Rossi E, Malgeri L, Stocchetti N, Galimberti S, Menon DK. Center-TBI participants and investigators. Management of arterial partial pressure of carbon dioxide in the first week after traumatic brain injury: results from the CENTER-TBI study. Intensive Care Med. 2021;47(9):961–73. 10.1007/s00134-021-06470-7. Epub 2021 Jul 24. 34302517 PMC8308080

[20] Lafave HC, Zouboules SM, James MA, Purdy GM, Rees JL, Steinback CD, Ondrus P, Brutsaert TD, Nysten HE, Nysten CE, Hoiland RL, Sherpa MT, Day TA. Steady-state cerebral blood flow regulation at altitude: interaction between oxygen and carbon dioxide. Eur J Appl Physiol. 2019;119(11–12):2529–44. 10.1007/s00421-019-04206-6. Epub 2019 Sep 26. 31559499

[21] Gonzalez-Garcia M, Maldonado D, Barrero M, Casas A, Perez-Padilla R, Torres-Duque CA. Arterial blood gases and ventilation at rest by age and sex in an adult Andean population resident at high altitude. Eur J Appl Physiol. 2020;120(12):2729–36. 10.1007/s00421-020-04498-z. Epub 2020 Sep 16. 32939642

[22] Godoy DA, Seifi A, Garza D, Lubillo-Montenegro S, Murillo-Cabezas F. Hyperventilation Therapy for Control of Posttraumatic Intracranial Hypertension. Front Neurol. 2017;8:250. 10.3389/fneur.2017.00250. 28769857 PMC5511895

[23] Li GH, Zhang YQ, Zhang HQ. Blood gas analysis of healthy people in Diqing Tibetan Autonomous Prefecture in Yunnan Province. Ann Palliat Med. 2021;10(1):285–291. 10.21037/apm-20-2206. Epub 2020 Dec 11. 33353353

[24] Greenspan L, McLellan BA, Greig H. Abbreviated Injury Scale and Injury Severity Score: a scoring chart. J Trauma. 1985;25(1):60 – 4. 10.1097/00005373-198501000-00010. 3965737

[25] Savitsky B, Givon A, Rozenfeld M, Radomislensky I, Peleg K. Traumatic brain injury: It is all about definition. Brain Inj. 2016;30(10):1194–200. Epub 2016 Jul 28. 27466967 10.1080/02699052.2016.1187290

[26] Foreman BP, Caesar RR, Parks J, Madden C, Gentilello LM, Shafi S, Carlile MC, Harper CR, Diaz-Arrastia RR. Usefulness of the abbreviated injury score and the injury severity score in comparison to the Glasgow Coma Scale in predicting outcome after traumatic brain injury. J Trauma. 2007;62(4):946 – 50. 10.1097/01.ta.0000229796.14717.3a. 17426553

[27] Vassallo J, Fuller G, Smith JE. Relationship between the Injury Severity Score and the need for life-saving interventions in trauma patients in the UK. Emerg Med J. 2020;37(8):502–7. 10.1136/emermed-2019-209092. Epub 2020 Jul 6. 32748796

[28] Marshall LF, Marshall SB, Klauber MR, Van Berkum Clark M, Eisenberg H, Jane JA, Luerssen TG, Marmarou A, Foulkes MA. The diagnosis of head injury requires a classification based on computed axial tomography. J Neurotrauma. 1992;9(Suppl 1):287–92. 1588618

[29] Charry JD, Navarro-Parra S, Solano J, Moscote-Salazar L, Pinzón MA, Tejada JH. Outcomes of traumatic brain injury: the prognostic accuracy of various scores and models. Neurol Neurochir Pol. 2019;53(1):55–60. 10.5603/PJNNS.a2018.0003. Epub 2019 Feb 11. 30742300

[30] Sadighi N, Talari H, Zafarmandi S, Ahmadianfard S, Baigi V, Fakharian E, Moussavi N, Sharif-Alhoseini M. Prediction of In-Hospital Outcomes in Patients with Traumatic Brain Injury Using Computed Tomographic Scoring Systems: A Comparison Between Marshall, Rotterdam, and Neuroimaging Radiological Interpretation Systems. World Neurosurg. 2023;175:e271–7. Epub 2023 Mar 21. 36958718 10.1016/j.wneu.2023.03.067

[31] Roozenbeek B, Lingsma HF, Lecky FE, Lu J, Weir J, Butcher I, McHugh GS, Murray GD, Perel P, Maas AI, Steyerberg EW, International Mission on Prognosis Analysis of Clinical Trials in Traumatic Brain Injury (IMPACT) Study Group. Prediction of outcome after moderate and severe traumatic brain injury: external validation of the International Mission on Prognosis and Analysis of Clinical Trials (IMPACT) and Corticoid Randomisation After Significant Head injury (CRASH) prognostic models. Crit Care Med. 2012;40(5):1609–17. 10.1097/CCM.0b013e31824519ce. Corticosteroid Randomisation After Significant Head Injury (CRASH) Trial Collaborators; Trauma Audit and Research Network (TARN). 22511138 PMC3335746

[32] Sun H, Lingsma HF, Steyerberg EW, Maas AI. External Validation of the International Mission for Prognosis and Analysis of Clinical Trials in Traumatic Brain Injury: Prognostic Models for Traumatic Brain Injury on the Study of the Neuroprotective Activity of Progesterone in Severe Traumatic Brain Injuries Trial. J Neurotrauma. 2016;33(16):1535–43. 10.1089/neu.2015.4164. Epub 2016 Feb 11. 26652051

[33] Steyerberg EW, Mushkudiani N, Perel P, Butcher I, Lu J, McHugh GS, Murray GD, Marmarou A, Roberts I, Habbema JD, Maas AI. Predicting outcome after traumatic brain injury: development and international validation of prognostic scores based on admission characteristics. PLoS Med. 2008;5(8):e165. 10.1371/journal.pmed.0050165. 18684008 PMC2494563

[34] Wilson JT, Pettigrew LE, Teasdale GM. Structured interviews for the Glasgow Outcome Scale and the extended Glasgow Outcome Scale: guidelines for their use. J Neurotrauma. 1998;15(8):573 – 85. 10.1089/neu.1998.15.573. 9726257

[35] Tewarie PKB, Beernink TMJ, Eertman-Meyer CJ, Cornet AD, Beishuizen A, van Putten MJAM, Tjepkema-Cloostermans MC. Early EEG monitoring predicts clinical outcome in patients with moderate to severe traumatic brain injury. Neuroimage Clin. 2023;37:103350. 10.1016/j.nicl.2023.103350. Epub 2023 Feb 14. 36801601 PMC9984683

[36] Yue JK, Kobeissy FH, Jain S, Sun X, Phelps RRL, Korley FK, Gardner RC, Ferguson AR, Huie JR, Schneider ALC, Yang Z, Xu H, Lynch CE, Deng H, Rabinowitz M, Vassar MJ, Taylor SR, Mukherjee P, Yuh EL, Markowitz AJ, Puccio AM, Okonkwo DO, Diaz-Arrastia R, Manley GT, Wang KKW. Neuroinflammatory Biomarkers for Traumatic Brain Injury Diagnosis and Prognosis: A TRACK-TBI Pilot Study. Neurotrauma Rep. 2023;4(1):171–83. 10.1089/neur.2022.0060. 36974122 PMC10039275

[37] Banoei MM, Lee CH, Hutchison J, Panenka W, Wellington C, Wishart DS, Winston BW, Clinical Network (CTRC). ; Canadian biobank, database for Traumatic Brain Injury (CanTBI) investigators, the Canadian Critical Care Translational Biology Group (CCCTBG), the Canadian Traumatic Brain Injury Research,. Using metabolomics to predict severe traumatic brain injury outcome (GOSE) at 3 and 12 months. Crit Care. 2023;27(1):295. 10.1186/s13054-023-04573-9. 37481590 PMC10363297

[38] Kalil AC, Metersky ML, Klompas M, Muscedere J, Sweeney DA, Palmer LB, Napolitano LM, O’Grady NP, Bartlett JG, Carratalà J, El Solh AA, Ewig S, Fey PD, File TM Jr, Restrepo MI, Roberts JA, Waterer GW, Cruse P, Knight SL, Brozek JL, the Infectious Diseases Society of America and the American Thoracic Society. Management of Adults With Hospital-acquired and Ventilator-associated Pneumonia: 2016 Clinical Practice Guidelines by. Clin Infect Dis. 2016;63(5):e61–e111. 10.1093/cid/ciw353. Epub 2016 Jul 14. Erratum in: Clin Infect Dis. 2017;64(9):1298. Erratum in: Clin Infect Dis. 2017;65(8):1435. Erratum in: Clin Infect Dis. 2017;65(12):2161. 27418577 PMC4981759

[39] Hooton TM, Bradley SF, Cardenas DD, Colgan R, Geerlings SE, Rice JC, Saint S, Schaeffer AJ, Tambayh PA, Tenke P, Nicolle LE. ; Infectious Diseases Society of America. Diagnosis, prevention, and treatment of catheter-associated urinary tract infection in adults: 2009 International Clinical Practice Guidelines from the Infectious Diseases Society of America. Clin Infect Dis. 2010;50(5):625 – 63. 10.1086/650482. 20175247

[40] Stevens DL, Bisno AL, Chambers HF, Dellinger EP, Goldstein EJ, Gorbach SL, Hirschmann JV, Kaplan SL, Montoya JG, Wade JC. ; Infectious Diseases Society of America. Practice guidelines for the diagnosis and management of skin and soft tissue infections: 2014 update by the Infectious Diseases Society of America. Clin Infect Dis. 2014;59(2):e10–52. 10.1093/cid/ciu444. Erratum in: Clin Infect Dis. 2015;60(9):1448. Dosage error in article text. 24973422

[41] Chaves F, Garnacho-Montero J, Del Pozo JL, Bouza E, Capdevila JA, de Cueto M, Domínguez MÁ, Esteban J, Fernández-Hidalgo N, Fernández Sampedro M, Fortún J, Guembe M, Lorente L, Paño JR, Ramírez P, Salavert M, Sánchez M, Vallés J. Diagnosis and treatment of catheter-related bloodstream infection: Clinical guidelines of the Spanish Society of Infectious Diseases and Clinical Microbiology and (SEIMC) and the Spanish Society of Spanish Society of Intensive and Critical Care Medicine and Coronary Units (SEMICYUC). Med Intensiva (Engl Ed). 2018 Jan-Feb;42(1):5–36. English, Spanish. 10.1016/j.medin.2017.09.012. 29406956

[42] McCrea MA, Giacino JT, Barber J, Temkin NR, Nelson LD, Levin HS, Dikmen S, Stein M, Bodien YG, Boase K, Taylor SR, Vassar M, Mukherjee P, Robertson C, Diaz-Arrastia R, Okonkwo DO, Markowitz AJ, Manley GT, TRACK-TBI Investigators; Adeoye O, Badjatia N, Bullock MR, Chesnut R, Corrigan JD, Crawford K, Duhaime AC, Ellenbogen R, Feeser VR, Ferguson AR, Foreman B, Gardner R, Gaudette E, Goldman D, Gonzalez L, Gopinath S, Gullapalli R, Hemphill JC, Hotz G, Jain S, Keene CD, Korley FK, Kramer J, Kreitzer N, Lindsell C, Machamer J, Madden C, Martin A, McAllister T, Merchant R, Ngwenya LB, Noel F, Nolan A, Palacios E, Perl D, Puccio A, Rabinowitz M, Rosand J, Sander A, Satris G, Schnyer D, Seabury S, Sherer M, Toga A, Valadka A, Wang, Yue JK, Yuh E, Zafonte R. Functional Outcomes Over the First Year After Moderate to Severe Traumatic Brain Injury in the Prospective, Longitudinal TRACK-TBI Study. JAMA Neurol. 2021;78(8):982–92. 10.1001/jamaneurol.2021.2043. 34228047 PMC8261688

[43] Maas AIR, Menon DK, Manley GT, Abrams M, Åkerlund C, Andelic N, Aries M, Bashford T, Bell MJ, Bodien YG, Brett BL, Büki A, Chesnut RM, Citerio G, Clark D, Clasby B, Cooper DJ, Czeiter E, Czosnyka M, Dams-O’Connor K, De Keyser V, Diaz-Arrastia R, Ercole A, van Essen TA, Falvey É, Ferguson AR, Figaji A, Fitzgerald M, Foreman B, Gantner D, Gao G, Giacino J, Gravesteijn B, Guiza F, Gupta D, Gurnell M, Haagsma JA, Hammond FM, Hawryluk G, Hutchinson P, van der Jagt M, Jain S, Jain S, Jiang JY, Kent H, Kolias A, Kompanje EJO, Lecky F, Lingsma HF, Maegele M, Majdan M, Markowitz A, McCrea M, Meyfroidt G, Mikolić A, Mondello S, Mukherjee P, Nelson D, Nelson LD, Newcombe V, Okonkwo D, Orešič M, Peul W, Pisică D, Polinder S, Ponsford J, Puybasset L, Raj R, Robba C, Røe C, Rosand J, Schueler P, Sharp DJ, Smielewski P, Stein MB, von Steinbüchel N, Stewart W, Steyerberg EW, Stocchetti N, Temkin N, Tenovuo O, Theadom A, Thomas I, Espin AT, Turgeon AF, Unterberg A, Van Praag D, van Veen E, Verheyden J, Vyvere TV, Wang KKW, Wiegers EJA, Williams WH, Wilson L, Wisniewski SR, Younsi A, Yue JK, Yuh EL, Zeiler FA, Zeldovich M, Zemek R. InTBIR Participants and Investigators. Traumatic brain injury: progress and challenges in prevention, clinical care, and research. Lancet Neurol. 2022;21(11):1004–60. 10.1016/S1474-4422(22)00309-X. Epub 2022 Sep 29. Erratum in: Lancet Neurol. 2022; 36183712 PMC10427240

[44] Steyerberg EW, Wiegers E, Sewalt C, Buki A, Citerio G, De Keyser V, Ercole A, Kunzmann K, Lanyon L, Lecky F, Lingsma H, Manley G, Nelson D, Peul W, Stocchetti N, von Steinbüchel N, Vande Vyvere T, Verheyden J, Wilson L, Maas AIR, Menon DK. ; CENTER-TBI Participants and Investigators. Case-mix, care pathways, and outcomes in patients with traumatic brain injury in CENTER-TBI: a European prospective, multicentre, longitudinal, cohort study. Lancet Neurol. 2019;18(10):923–934. 10.1016/S1474-4422(19)30232-7. 31526754

[45] Zuckerman DA, Giacino JT, Bodien YG, Outcome?. J Neurotrauma. 2022;39(13–14):1010–2. 10.1089/neu.2021.0356. Epub 2021 Dec 22. 34861770 PMC9248332

[46] Claassen J, Doyle K, Matory A, Couch C, Burger KM, Velazquez A, Okonkwo JU, King JR, Park S, Agarwal S, Roh D, Megjhani M, Eliseyev A, Connolly ES, Rohaut B. Detection of Brain Activation in Unresponsive Patients with Acute Brain Injury. N Engl J Med. 2019;380(26):2497–2505. 10.1056/NEJMoa1812757. 31242361

[47] Cooper DJ, Rosenfeld JV, Murray L, Arabi YM, Davies AR, Ponsford J, Seppelt I, Reilly P, Wiegers E, Wolfe R, DECRA Trial Investigators and the Australian and New Zealand Intensive Care Society Clinical Trials Group. Patient Outcomes at Twelve Months after Early Decompressive Craniectomy for Diffuse Traumatic Brain Injury in the Randomized DECRA Clinical Trial. J Neurotrauma. 2020;37(5):810–6. 10.1089/neu.2019.6869. 32027212 PMC7071071

[48] Kolias AG, Adams H, Timofeev IS, Corteen EA, Hossain I, Czosnyka M, Timothy J, Anderson I, Bulters DO, Belli A, Eynon CA, Wadley J, Mendelow AD, Mitchell PM, Wilson MH, Critchley G, Sahuquillo J, Unterberg A, Posti JP, Servadei F, Teasdale GM, Pickard JD, Menon DK, Murray GD, Kirkpatrick PJ, Hutchinson PJ. RESCUEicp Trial Collaborators. Evaluation of Outcomes Among Patients With Traumatic Intracranial Hypertension Treated With Decompressive Craniectomy vs Standard Medical Care at 24 Months: A Secondary Analysis of the RESCUEicp Randomized Clinical Trial. JAMA Neurol. 2022;79(7):664–71. 10.1001/jamaneurol.2022.1070. 35666526 PMC9171657

[49] Toro C, Hatfield J, Temkin N, Barber J, Manley G, Ohnuma T, Komisarow J, Foreman B, Korley FK, Vavilala MS, Laskowitz DT, Mathew JP, Hernandez A, Sampson J, James ML, Raghunathan K, Goldstein BA, Markowitz AJ, Krishnamoorthy V, TRACK-TBI Investigators. Risk Factors and Neurological Outcomes Associated With Circulatory Shock After Moderate-Severe Traumatic Brain Injury: A TRACK-TBI Study. Neurosurgery. 2022;91(3):427–36. 10.1227/neu.0000000000002042. Epub 2022 May 24. 35593705 PMC10553078

[50] Kowalski RG, Hammond FM, Weintraub AH, Nakase-Richardson R, Zafonte RD, Whyte J, Giacino JT. Recovery of Consciousness and Functional Outcome in Moderate and Severe Traumatic Brain Injury. JAMA Neurol. 2021;78(5):548–57. 10.1001/jamaneurol.2021.0084. 33646273 PMC7922241

[51] Watanitanon A, Lyons VH, Lele AV, Krishnamoorthy V, Chaikittisilpa N, Chandee T, Vavilala MS. Clinical Epidemiology of Adults With Moderate Traumatic Brain Injury. Crit Care Med. 2018;46(5):781–7. 29369057 10.1097/CCM.0000000000002991 PMC5899009

[52] Robba C, Galimberti S, Graziano F, Wiegers EJA, Lingsma HF, Iaquaniello C, Stocchetti N, Menon D, Citerio G. CENTER-TBI ICU Participants and Investigators. Tracheostomy practice and timing in traumatic brain-injured patients: a CENTER-TBI study. Intensive Care Med. 2020;46(5):983–94. Epub 2020 Feb 5. 32025780 10.1007/s00134-020-05935-5 PMC7223805

[53] Robba C, Graziano F, Rebora P, Elli F, Giussani C, Oddo M, Meyfroidt G, Helbok R, Taccone FS, Prisco L, Vincent JL, Suarez JI, Stocchetti N, Citerio G. ; SYNAPSE-ICU Investigators. Intracranial pressure monitoring in patients with acute brain injury in the intensive care unit (SYNAPSE-ICU): an international, prospective observational cohort study. Lancet Neurol. 2021;20(7):548–558. 10.1016/S1474-4422(21)00138-1. 34146513

[54] de Cássia A, Vieira R, Silveira JCP, Paiva WS, de Oliveira DV, de Souza CPE, Santana-Santos E, de Sousa RMC. Prognostic Models in Severe Traumatic Brain Injury: A Systematic Review and Meta-analysis. Neurocrit Care. 2022;37(3):790–805. 10.1007/s12028-022-01547-7. Epub 2022 Aug 9. 35941405

[55] Cremer OL, Moons KG, van Dijk GW, van Balen P, Kalkman CJ. Prognosis following severe head injury: Development and validation of a model for prediction of death, disability, and functional recovery. J Trauma. 2006;61(6):1484–91. 10.1097/01.ta.0000195981.63776.ba. 16983303

[56] MRC CRASH Trial Collaborators, Perel P, Arango M, Clayton T, Edwards P, Komolafe E, Poccock S, Roberts I, Shakur H, Steyerberg E, Yutthakasemsunt S. Predicting outcome after traumatic brain injury: practical prognostic models based on large cohort of international patients. BMJ. 2008;336(7641):425–9. 10.1136/bmj.39461.643438.25. Epub 2008 Feb 12. 18270239 PMC2249681

[57] Johnston AJ, Steiner LA, Gupta AK, Menon DK. Cerebral oxygen vasoreactivity and cerebral tissue oxygen reactivity. Br J Anaesth. 2003;90(6):774 – 86. 10.1093/bja/aeg104. 12765894

[58] Schalén W, Messeter K, Nordström CH. Cerebral vasoreactivity and the prediction of outcome in severe traumatic brain lesions. Acta Anaesthesiol Scand. 1991;35(2):113 – 22. 10.1111/j.1399-6576.1991.tb03258.x. 1902616

[59] Bélanger M, Allaman I, Magistretti PJ. Brain energy metabolism: focus on astrocyte-neuron metabolic cooperation. Cell Metab. 2011;14(6):724 – 38. 10.1016/j.cmet.2011.08.016. 22152301

[60] Launey Y, Fryer TD, Hong YT, Steiner LA, Nortje J, Veenith TV, Hutchinson PJ, Ercole A, Gupta AK, Aigbirhio FI, Pickard JD, Coles JP, Menon DK. Spatial and Temporal Pattern of Ischemia and Abnormal Vascular Function Following Traumatic Brain Injury. JAMA Neurol. 2020;77(3):339–49. 10.1001/jamaneurol.2019.3854. 31710336 PMC6865302

[61] Battisti-Charbonney A, Fisher J, Duffin J. The cerebrovascular response to carbon dioxide in humans. J Physiol. 2011;589(Pt 12):3039–48. 10.1113/jphysiol.2011.206052. Epub 2011 Apr 26. 21521758 PMC3139085

[62] Yoshihara M, Bandoh K, Marmarou A. Cerebrovascular carbon dioxide reactivity assessed by intracranial pressure dynamics in severely head injured patients. J Neurosurg. 1995;82(3):386 – 93. 10.3171/jns.1995.82.3.0386. 7861215

[63] Huijben JA, Volovici V, Cnossen MC, Haitsma IK, Stocchetti N, Maas AIR, Menon DK, Ercole A, Citerio G, Nelson D, Polinder S, Steyerberg EW, Lingsma HF, van der Jagt M. CENTER-TBI investigators and participants. Variation in general supportive and preventive intensive care management of traumatic brain injury: a survey in 66 neurotrauma centers participating in the Collaborative European NeuroTrauma Effectiveness Research in Traumatic Brain Injury (CENTER-TBI) study. Crit Care. 2018;22(1):90. 10.1186/s13054-018-2000-6. 29650049 PMC5898014

